# Standard setting anchor statements: a double cross-over trial of two different methods

**DOI:** 10.15694/mep.2021.000032.1

**Published:** 2021-02-03

**Authors:** Steven Burr, Theresa Martin, James Edwards, Colin Ferguson, Kerry Gilbert, Christian Gray, Adele Hill, Joanne Hosking, Karen Johnstone, Jolanta Kisielewska, Chloe Milsom, Siobhan Moyes, Ann Rigby-Jones, Iain Robinson, Nick Toms, Helen Watson, Daniel Zahra

**Affiliations:** 1 Peninsula Medical School; 2 School of Clinical Medicine

**Keywords:** Angoff, standard setting, assessment

## Abstract

This article was migrated. The article was marked as recommended.

**Context:** We challenge the philosophical acceptability of the Angoff method, and propose an alternative method of standard setting based on how important it is for candidates to know the material each test item assesses, and *not* how difficult it is for a subgroup of candidates to answer each item.

**Methods:** The practicalities of an alternative method of standard setting are evaluated here, for the first time, with direct comparison to an Angoff method. To negate bias due to any leading effects, a prospective cross-over design was adopted involving two groups of judges (n=7 and n=8), both of which set the standards for the same two 100 item multiple choice question tests, by the two different methods.

**Results:** Overall, we found that the two methods took a similar amount of time to complete. The alternative method produced a higher cut-score (by 12-14%), and had a higher degree of variability between judges’ cut-scores (by 5%). When using the alternative method, judges reported a small, but statistically significant, increase in their confidence to decide accurately the standard (by 3%).

**Conclusion:** This is a new approach to standard setting where the quantitative differences are slight, but there are clear qualitative advantages associated with use of the alternative method.

## Introduction

The Angoff method is widely used in the setting of standards for high-stakes medical assessments. Its proponents argue that its conceptual basis, estimating the performance of a group of borderline candidates, results in a reasonable standard. In practice, however, the assumptions of this conceptual ideal are difficult to realise. We have previously made the case that the Angoff method is conceptually flawed, outlining, among other issues, the difficulties with identifying a borderline group and estimating their performance ( Burr *et al*., 2017). Here we present and evaluate an alternative approach that emphasises the importance of knowledge for all candidates, rather than the difficulty in answering experienced by a few. Importance of each item was rated using three criteria defined in the description of the technique below. The Angoff method is the most widely used and researched method of criterion standard setting ( Talente *et al*., 2003, Stern *et al*. 2005, Bouriscot and Roberts, 2006, Elfaki and Salih, 2015), and the modified method has good inter-rater and moderate test-retest reliability ( George *et al.,* 2006). Angoff has lost some ground in clinical settings ( Smee and Blackmore, 2001), and more recently approaches to standard setting which better protect patient safety have been proposed ( Yudkowsky *et al.,* 2014, Barsuk *et al*., 2018). However, the Angoff method retains its primacy for standard setting even the most challenging and high-stakes of knowledge assessments ( Verhoeven *et al*., 1999, Elfaki and Salih, 2015, Karcher, 2019, Marcham, 2019). Therefore, we chose to compare an alternative with the Angoff method, and to evaluate the resulting scores as well as to undertake a rudimentary cost utility analysis. The alternative method ( Burr *et al*., 2017), relies on explicit judgements of how important each item’s content is for all candidates. This is in contrast to both the Angoff method, which, if judges behave as instructed, relies on judgements of how difficult each item would be for a borderline candidate ( Angoff, 1971), and the Ebel method, which relies on combining judgements of both difficulty and importance ( Ebel, 1972). Yudkowsky and colleagues have argued that if it is important to demonstrate mastery of clinical skills before progressing to the next stage of learning or practice, then a low level of achievement does not lessen that importance nor provide cause to lower the standard but rather identifies a gap that needs to be closed ( Yudkowsky *et al*., 2015). We contend that the same is true for the development of understanding of the key concepts that contribute to a sound and usable medical knowledge ( Neve *et al*., 2019). Thus, with the alternative method presented here, a standard is set based on what it is important to achieve, and not how difficult it is to achieve it, and for what all candidates should achieve, and not what a subgroup of candidates would achieve. Whilst judges taking part in other methods may implicitly or subconsciously incorporate other elements such as importance, difficulty, or curriculum delivery, these facets are not explicitly part of the process and their occurrence only serves to highlight conceptual difficulties and pragmatic issues with the methods. The alternative method explicitly seeks to elicit judgements of different facets of importance, and provides a clearly delineated method for combining them.

We postulate that the desirable qualities of a standard setting method are an extension of those that apply to assessments in general. There are many important values and principles for assessment design ( Norcini, 2003; Race *et al*., 2005). However, assessments should foremost be valid, reliable, discriminating, transparent and fair ( Burr, 2009; Scott *et al*., 2014). We further argue that they also need to be reasonable and acceptable to stakeholders. The aims of the current study were to evaluate the practicality of implementing our proposed alternative method, compare the standard derived using this new alternative method against a standard set using an Angoff method, and assess the judges’ acceptability of implementing each method.

## Methods

### Study design

Two groups of judges were recruited, A ( *n*=7) and B ( *n*=8), each with a similar mix of areas of expertise. Judges were all teaching staff with substantial experience of the breadth and depth of content knowledge expected of students, in part due facilitating enquiry based learning in a programme that is both vertically and horizontally integrated. All judges received training, including scoring questions using both methods guided by an instruction sheet, and had the opportunity to contribute to the refinement of both methods. Judges were allocated to groups A and B randomly, but stratified to take account of discipline expertise and prior experience of the Angoff process. Two summative Single-Best-Answer Multiple Choice Question tests, T1 and T2, each comprising 100 negatively marked items focussed on end of year one medical knowledge (encompassing all aspects of the healthy individual from a biopsychosocial perspective), were standard set by two methods by both groups of judges.

Method 1 used a percentage based Angoff approach, and Method 2 was our proposed alternative, as described by Burr *et al*. (2017). Judges were also asked to rate their own confidence in the accuracy of their decisions for each item during both methods on a three-point scale ranging from 1 (lowest) to 3 (highest) confidence. They were also asked to record both the time taken and their satisfaction with each method, and provided space for open-ended comments concerning their thoughts on the process of each method. During the moderation meetings, comments about the standard setting process were also recorded to provide further qualitative data.

**Figure 1.**
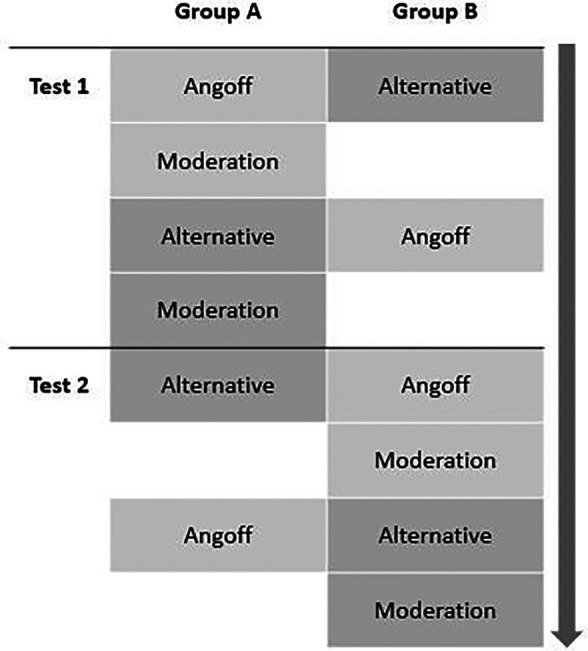
Schematic representation of the study design

Judges were sent questions to standard set three weeks before meeting and asked to return their ratings one week before meeting. There was a three week interval between each standard setting exercise. A double cross-over method design was adopted to prevent the possibility that familiarity with the questions could have a leading effect on judges’ ratings. The study design is detailed in Figure 1.

The relevant Faculty Ethics Committee was approached and confirmed that study-specific approval was not required. All judges are co-authors. The first and last authors were not judges, but led and analysed the study data respectively.

### Summary of alternative method

For every item, each judge is required to rate three elements: Links, Frequency, and Consequences. These elements combined indicate a level of item importance. Links (L) refers to how much the knowledge provides a fundamental basis and foundation for other concepts (conditional knowledge within the same discipline and for other disciplines; bearing in mind any other compensatory knowledge). Frequency (F) refers to how often the knowledge is required during the next stage of study. Consequences (C) refers to the impact if the knowledge was misunderstood and misapplied, or required but not known during the next stage (stage refers to a year of study or a point in the formal progression of a candidate).

Each rating is made on a scale of 1 to 3 (1 representing the lowest rating, 3 representing the highest rating). The anchor statement stresses: When deciding each rating, you should consider the information in all aspects of the item (including the vignette and answer options), and its importance. Importance is judged relative to the purpose of the test as a gate to ensure fitness to progress to the next stage of study. Unless setting a standard for a final assessment, judges should try not to be influenced by the impact a lack of knowledge would have in clinical practice once qualified. This is in contrast to the Angoff anchor statement which states: Your score indicates the average percentage that ‘just-passing’ (minimally competent) candidates would achieve for that question ( Hess *et al*., 2007).

For the alternative method, the concept of item difficulty for specific candidates should be disregarded. However, the ability to distinguish between ‘easy’ and ‘plausible distractors should be taken into consideration when determining the importance of all the knowledge needed to answer the item correctly as a whole. The distractors factor into the judgement in the alternative method because it is necessary to consider whether it’s important that candidates know the difference between the distractors. One set of distractors would quite possibly result in a different importance rating to another set of distractors for the same question. Thus, the number of factors and degree of synthesis required to answer an item correctly may be considered to represent difficulty, but should instead be included in consideration of importance. For example, rating an item a ‘1’ may indicate the knowledge is intended to stretch candidates (what they could know); ‘2’ may indicate the knowledge is important (what they should know); and ‘3’ may indicate the knowledge is essential (what they would know).

In order to derive a cut-score from element scores, each of the elements are summed with equal weighting (L+F+C), converted to a percentage, corrected for the lack of a zero point, and averaged across items within judges, then across judges to obtain overall cut-score.

**Figure 2.**
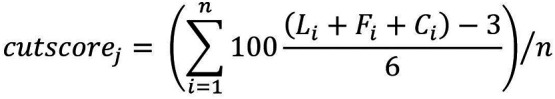
Equation to calculate cutscores*

*Cutscore for each judge, j, where n is the number of items, i.

### Comparisons to Angoff

In order to address our research aims, an analysis of variance was used to compare the standards derived using the Angoff method with the standards derived using the alternative method. Pearson correlation coefficients were calculated as a measure of the relationship between item-level standards generated by the Angoff and alternative methods (derived from a combination of L, F, and C ratings as described above), both after judges’ initial ratings (Phase 1), and after a moderation meeting (Phase 2). Correlations were not separated by Test as the relationship of interest is between item-level standards, and items may appear in any test in practice.

Descriptive statistics reflecting the distribution of individual judge’s cut-scores (an average of each of their item ratings) for each method before and after moderation sessions are also provided as a measure of the variability of cut-scores generated by each method and the impact of discussion in moderation meetings. Inter-rater reliability was also evaluated for each method by calculating intra-class correlation coefficients of agreement between individual judges’ item-level standards before and after moderation meetings for each method.

## Results/Analysis

### Comparison of cut-scores between Angoff and alternative method standards

The cut-scores generated by each phase of the Angoff and alternative methods are shown below ( Table 1). The cut-scores generated for each data collection point are shown in Table 2. These values provide an indication of the variation in cut-scores generated by each method, as well as an indication of the impact of moderation meetings.

**Table 1.** Overall Phase One and Phase Two cut-scores *

	Phase One		Phase Two	
Method	Mean(%)	SD(%)	Mean (%)	SD(%)
Angoff	40.50	22.28	40.59	21.53
Alternative	52.96	27.65	54.67	26.80

* Overall Phase One (individual ratings) and Phase Two (after a moderation meeting) cut-scores derived using the Angoff and alternative methods (n=15 judges).

**Table 2.** Cut-scores by Group, Test, and Phase *

		Angoff		Alternative		Phase Difference		Method Difference	
Group	Test	Phase 1	Phase 2	Phase 1	Phase 2	Angoff	Alternative	Phase 1	Phase 2
A	1	44.63	42.60	58.81	65.55	-2.03	6.74	14.18	22.95
A	2	40.21	---	61.79	---	---	---	21.57	---
B	1	38.44	---	46.10	---	---	---	7.67	---
B	2	39.22	38.88	46.96	45.15	-0.34	-1.81	7.74	6.27

* Cut-scores (%) by Group, Test, and Phase, including difference in cut-scores between methods at each phase and between phases for each method (n=7 judges in Group A, and 8 judges in Group B).

Pearson correlation coefficients suggest that the individual item ratings generated by each phase of each method are positively correlated ( *p*<0.001). However, the individual item ratings generated by each method are not ( *p*>0.05). This pattern of correlations suggests that item difficulty (of which the Angoff ratings are a measure) is not necessarily associated with the importance of the content assessed by each item (of which the alternative method ratings provides a measure).

**Table 3.** Pearson correlation coefficients for the correlations between item ratings *

p-values		Angoff (Phase2)	Alternative (Phase1)	Alternative (Phase2)
Angoff (Phase2)	r	0.947	0.245	0.336
	p	<0.001	0.192	0.221
Alternative (Phase1)	r		-0.088	0.088
	p		0.775	0.775
Alternative (Phase2)	r			0.846
	p			<0.001

* Pearson correlation coefficients (and p-values) for the correlations between item ratings (averaged across Judges) derived from each method at each phase.

A two Method (Angoff, Alternative; within-participants) by two Phase (One, Two; within participants) by 15 Judges (individual Judges; between-participants) analysis of variance was conducted on item ratings. Judges showed a statistically significant main effect of Method, *F*(1,1287)=220.863, *p*<0.001, but not Phase, *F*(1,1287)=3.472, *p*=0.063. There was also a statistically significant main effect of Judge, *F*(1,1287)=14.965, *p*<0.001. The Angoff method produced significantly lower standards than the alternative, and Phase Two standards were marginally higher than Phase One.

Inter-rater reliability was assessed by calculating intra-class correlation coefficients, both within Groups and Tests by Method and Phase across item-standards from each Judge. The results of these analyses are shown in Table 4. All subsets of item ratings show significant absolute agreement between raters, and absolute agreement between raters increased between Phase One and Phase Two within both methods. Intra-class correlation coefficients were modelled for absolute agreement rather than consistency between item-standards across Judges.

**Table 4.** Intra-class Correlation Coefficients of agreement between item judgements *

		Angoff	Alternative				
Group	Test	Phase 1	Phase 2	Difference	Phase 1	Phase 2	Difference
A	1	0.287	0.489	0.202	0.203	0.389	0.186
A	2	0.266	---	---	0.126	---	---
B	1	0.097	---	---	0.196	---	---
B	2	0.107	0.260	0.153	0.191	0.377	0.186
Mean		0.189	0.375	0.178	0.179	0.383	0.186

* Intra-class Correlation Coefficients (two-way random model) of agreement between item judgements within Groups and Tests by Method and Phase. All statistically significant at *p*<0.001.

### Confidence in Angoff and Alternative Judgements

Table 5 shows the mean confidence ratings of each judge, across all items, by Group, Test, Phase, and Method. Although there was a statistically significant positive correlation between Angoff and Alternative confidence ratings ( *r*=0.458, *p*<0.001), a 2 Method (Angoff, Alternative; within participants) by 15 Judge (individual Judges; between-participants) ANOVA on Phase One confidence ratings showed that the alternative method elicited significantly greater confidence ratings ( *M*=2.02, *SD*=0.73) than the Angoff method ( *M*=1.92, *SD*=0.77), *F*(1,2985)=-46.644, *p*<.001.

**Table 5.** Mean confidence ratings (out of 3) by Group and Test across methods (Phase 1)

		Angoff (Phase 1)		Alternative (Phase 2)	
Group	Test	M	SD	M	SD
A	1	1.98	0.73	1.86	0.77
A	2	1.88	0.82	2.16	0.69
B	1	1.91	0.77	2.02	0.72
B	2	1.91	0.77	2.02	0.72
Overall		1.92	0.77	2.02	0.73

### Feedback from judges

There was a consensus that the Links element represented the number of other domains the concept or concepts in the item were connected to. There was also consensus that Frequency represented the number of times the concept was likely to be encountered in the next stage of the programme. Whereas, Consequences represented the impact if the content was not understood in the next stage of the programme. However, it was thought that all three overlapped to some extent. There was the possibility for deducing that high Frequency may correspond to low Consequence, due to opportunities for remediation. In the moderation meetings, it was necessary to remind judges to: (1) completely disregard all ideas of difficulty, (2) ensure that they considered and factored in the importance of distractors and wider applications of the content (in addition to the specific intrinsic question and answer, and, (3) consider the importance of the question content for the remainder of the programme and not for clinical practice after qualification (as this was an end of year one test).

### Qualitative comments about alternative method

As part of Phase One, where judges provided their individual item ratings, and Phase Two during moderation meetings, they were given the opportunity to provide qualitative feedback on the processes and methods. These comments focussed on the shift from difficulty to importance, and the change in perspective from the familiar Angoff process. Examples of judges’ comments are provided below.

• “provides a form of quality control for teaching content that we don’t get from Angoff.”
• “made me challenge my assumptions and think about the questions in a different way.”
• “may encourage me to change the way questions are written, to maximise the impact for importance of all 3 elements of importance (e.g. to make vignettes more explicit).”
• “encourages staff to learn more about how their discipline fits in to the whole curriculum.”
• “takes less time than Angoff, even though we were less familiar with it.”

Whilst these comments are based on a small sample of judges, there was general agreement amongst judge participants, which would suggest there is some weight in their overall sentiment. Thus, these reflections provide additional support for the alternative method and additional context to the quantitative findings.

### Time taken

Judges were also asked to record how long Phase One of each method took them to complete, and the meeting chairs recorded the duration of the moderation meetings. Timings showed that Phase One of the alternative method took on average 27 minutes less time to complete that Phase One of the Angoff method (Alternative: *M*=108, *SD*=42, Angoff: *M*=135, *SD*=41 minutes; *t*(15)=2.791, *p*=0.014). The moderation meetings for the alternative method were also shorter ( *M*=121, *SD*=28 minutes) than those for the Angoff method ( *M*=137, *SD*=31 minutes), though this difference was not statistically significant, *t*(14)=1.065, *p*=0.305. However, although small, these differences may be significant time savings to individuals in practical terms, and would certainly represent large cumulative time savings over multiple standard setting processes.

## Discussion

### Comparison of scores between the two methods

Is there an assumption in the Angoff method that all items are of equal importance? Angoff focusses on rating item difficulty irrespective of importance. Similarly, the alternative method makes an assumption that all items are of equal difficulty, as it focusses on importance irrespective of difficulty. With the Angoff method, larger numbers of more difficult items reduces the cut-score (as borderline candidates find it more difficult) (Bed-David, 2000). In contrast, with the alternative method, larger numbers of low-importance items reduces the cut-score. It follows that a high Angoff cut-score reflects a greater number of less difficult items, and a higher alternative cut-score reflects a greater number of high-importance items. Including more low-importance items reduces the cut-score, but this does not necessarily mean the assessment is easier to pass as we don’t know what the item difficulty is. How easy it is does not matter; what matters is whether candidates can do what is required, i.e. whether they know what is important. This paradigm shift is the crux of the conceptual difficulty in describing one method in terms of the other, and the associated interpretation of the resultant cut-score. The Angoff method provides a measure of the difficulty of the test items, and what the *borderline* candidates *would* know. The alternative method provides a measure of the importance of the content assessed by the test items, and what *all* candidates *should* know.

There is a tension between trying to set a standard to identify candidates who should just pass, and defining a range of abilities, when using test items of varying importance. Excellence cannot be defined by how much of the essential content someone knows, as they should ideally know all the essential content in order to pass. Thus, excellence should be defined as a score that represents development beyond the required standard for the particular stage of study, i.e., reaching into the knowledge that might reasonably be expected in subsequent years. A stretch objective tests more detailed information or understanding than is needed for competence, and thus lowers the alternative method cut-score (and can also be expected to be inherently more difficult if the content doesn’t receive as much emphasis in the syllabus). It does not matter if a test includes a higher proportion of less important content and thus has a lower cut score; what matters is that tests are set to test the necessary learning outcomes and that candidates are given fair opportunity to demonstrate they have met the standards that are required. However, the less important the content the more compensation is possible, reducing the need to answer the more important questions correctly to meet the standard. More questions of lower importance also directly increases the scope for demonstrating excellence, where demonstrating excellence depends on achieving more specialist knowledge stretch objectives; and achieving higher overall scores relative to the cut-score. Naturally, the more essential content there is in an assessment the higher the cut-score will be. This highlights the need to have the assessment content aligned to the syllabus and aims of the curriculum.

### Acceptability of conceptualisation, and the need to agree weightings

It is logical to assume that expert knowledge may influence perceptions of importance ( Clauser *et al*., 2016), but there are several elements of importance that can be considered. Advocacy for the importance of each judge’s own area of expertise in content knowledge may be moderated, to some extent by rating each question in more than one element of importance, for example, the links to other concepts, the frequency with which the concept is encountered, and the consequences of not understanding a concept ( Neve *et al*., 2019).

Given the paradigm shift from difficulty to importance involved in conceptualising the alternative method, it is notable that the time taken to complete Phase One and Phase Two of the standard setting process tended to be shorter than for the more familiar Angoff method. It may be easier to conceptualise and keep ratings focussed on what candidates need to know if the judges don’t need to simultaneously keep in mind a reference group of candidates and estimate their performance in percentage terms. The use of equal weighting, or an Ebel-type grid, removes the need to decide a score of 0-100.

All judges agreed that it was philosophically and pedagogically more acceptable to set a pass-fail threshold or cut-score based on “how *important* it is for *all* candidates to know something” (at the particular point of assessment), rather than rate the difficulty of answering each item correctly for a hypothetical, subjectively-defined subgroup of candidates. For example, a CPR OSCE station wouldn’t be set on how difficult it is for the average borderline candidate to pass, but on how important it is for any and all candidates to be able to successfully demonstrate the required skills.

Our initial approach has been to consider each of the three elements of importance as equally important. This is in contrast to standard-setting patient safety where patient safety is an issue and thus full and equal compensation may not be appropriate ( Downing and Yudkowsky, 2009). To ameliorate for this, the method could reasonably be used with quotas and separate cut-scores for each element although this might undermine our attempt to limit judge bias and would need to be tested in future studies. In addition, our alternative method could easily be adapted to different contexts. For example, a foundation programme may require a greater emphasis on Links but be less applied and thus have lower scores for Frequency and Consequences, and thus be weighted accordingly. Depending on the subject context, more (or less) than three elements of importance may be appropriate, the weighting between them need not be equal, and the elements of importance could themselves be different.

All of the items came from a bank that were written in anticipation of being used for an Angoff process. Use of the alternative method for standard setting may influence the writing of future questions to improve their alignment with importance. Similarly learning outcomes may reemphasise expectations and be utilised by staff and students differently, focussing efforts to increase performance based on importance. As judges are more confident in the accuracy of the higher standard, then perhaps an interim period is needed which includes application of an offset value to ensure the practical acceptability of the final cut-score. If the alternative method sets a high cut-score, because candidates need to know more important things to pass, it may increase the failure rate. If something is important then the candidates need to know it, and those who don’t know what they should may pass if the standard is set using Angoff. Do we stand our ground and argue that the cut-scores have been set too low historically? Instead, it may be prudent to supplement the method with a Hofstee moderation as a safeguard ( Hofstee, 1983). This could be as a temporary interim measure until teaching and question writing develop to reflect the focus on importance rather than difficulty where the alternative method is applied to programmes with existing, historically difficulty-focussed assessments.

### Cost utility evaluation

The alternative method was found to be: (1) valid, in as far as judges agreed on and favoured the focus on importance-based pedagogy; (2) reliable, with respect to consistency and agreement within and between judges’ ratings; (3) discriminating, as candidates who don’t know the important content will fail, irrespective of difficulty; (4) transparent, as it is possible for standard setting scores to be mapped to learning outcomes and correlated with performance; (5) fair, as the focus is moved from borderline candidates to the standard required by all candidates; and (6) reasonable as feedback confirmed it was acceptable to staff both conceptually and in terms of time commitment.

## Conclusion

Standard setting procedures should be chosen to produce the most appropriate decision distribution in the context to which they are applied. With the Angoff method, difficult items lead to a low cut-score, even if it is more important to have learnt the content of those items. The Angoff method favours candidates who can answer difficult items regardless of their importance, and thus discriminates against those who can answer important items that are less difficult. The alternative method has several potential advantages over the Angoff method: (1) it provides an integral quality assurance check that a test contains what is important; (2) the standard setting scores could be validated by direct correlation with the performance of all candidates, not just a borderline group that can only be defined by the test; (3) ranking for elements of importance could become a feedback stream for candidates; (4) items could be written or modified to fit the matrix (to give specific scores in the different elements of importance); and (5) a sufficiently detailed searchable curriculum map could be used to semi-automatically populate standard setting ratings. It follows that a map of the occurrence of concepts could be used to check the spread, order, and spiralling of knowledge throughout a curriculum.

## Take Home Messages

• Our quantitative analyses do not reveal one method as statistically superior to the other.
• Our qualitative findings indicate that the alternative method is philosophically better justified with certain conceptual, and potential practical, advantages.

## Notes On Contributors

**Steven A Burr** is a Professor in Medical Education at Peninsula Medical School, Faculty of Health, University of Plymouth, UK. ORCID ID: https://orcid.org/0000-0002-0222-605X

**Theresa Martin** (nee Compton) is a Lecturer in Biomedical Sciences at Peninsula Medical School, Faculty of Health, University of Plymouth, UK.

**James Edwards** is a Lecturer in Biomedical Sciences at Peninsula Medical School, Faculty of Health, University of Plymouth, UK.

**Colin Ferguson** is a Consultant Intensivist, University Hospitals NHS Trust, Plymouth, UK.

**Kerry Gilbert** is an Associate Professor of Medical Education and Medical Sciences at Peninsula Medical School, Faculty of Health, University of Plymouth, UK.

**Christian P Gray** is a Senior Lecturer, St Lucia Clinical Unit, Faculty of Medicine, University of Queensland, Australia, and was previously a Lecturer at Peninsula Medical School.

**Adele Hill** is an Associate Professor (Education) at Peninsula Medical School, and Associate Dean for Teaching and Learning in the Faculty of Health, University of Plymouth, UK.

**Joanne Hosking** is a Senior Research Fellow at Peninsula Medical School, Faculty of Health, University of Plymouth, UK.

**Karen Johnstone** is an Associate Professor of Genetics and Genomics at Peninsula Medical School, Faculty of Health, University of Plymouth, UK.

**Jolanta Kisielewska** is an Associate Professor of Medical Education at Peninsula Medical School, Faculty of Health, University of Plymouth, UK.

**Chloe Milsom** is a Lecturer in Biomedical Sciences at Peninsula Medical School, Faculty of Health, University of Plymouth, UK.

**Siobhan Moyes** is a Lecturer in Anatomy at Peninsula Medical School, Faculty of Health, University of Plymouth, UK.

**Ann E Rigby-Jones** is a Lecturer in Pharmacology at Peninsula Medical School, Faculty of Health, University of Plymouth, UK.

**Iain M Robinson** is an Associate Professor (Reader) in Neurosciences at Peninsula Medical School, Faculty of Health, University of Plymouth, UK.

**Nick Toms** is an Associate Professor and Deputy Head of School for Peninsula Medical School, Faculty of Health, University of Plymouth, UK.

**Helen R Watson** is an Associate Professor of Bioscience at Peninsula Medical School, Faculty of Health, University of Plymouth, UK.

**Daniel Zahra** is a Lecturer in Assessment Psychometrics at the Peninsula Schools of Medicine and Dentistry, Faculty of Health, University of Plymouth, UK, with an interest in psychology and interprofessional education. ORCID ID: https://orcid.org/0000-0001-6534-6916
