# Supplementary material for: Standard setting anchor statements: a double cross-over trial of two different methods
Source: MedEdPublish (2016). 2021 Feb 3;10:32. [Version 1] doi: 10.15694/mep.2021.000032.1 (PMC10939605; doi:10.15694/mep.2021.000032.1)
Supplement: Peer review history

# Reviewer report: Reviewer response for version 1

**DOI:** 10.21956/mep.18847.r26864

**Published:** 2021-05-10

**Authors:** Ken Masters

**Affiliations:** 1 Sultan Qaboos University

This review has been migrated. The reviewer awarded 4 stars out of 5 An interesting piece of research on a standard-setting method as a direct challenge to the Angoff method.I don’t really agree with the authors’ premise (especially the statement “How easy it is does not matter; what matters is whether candidates can do what is required, i.e. whether they know what is important.”). Personally, I think it’s a combination of the two. (Because it is possible to set an easy question on an important topic, and so, a student may not have real mastery of the important topic, even though they got the answer correct). As a reviewer of this paper, however, I think this is a novel approach, so my personal feelings are not relevant. I hope that others will take it further with experiments and tests.“It does not matter if a test includes a higher proportion of less important content and thushas a lower cut score” (and the sentences that follow). I’m not sure if I have misunderstood what the authors are arguing, but I would argue that, with their method, the low cut score would actually be a useful alert that the test might be trivial, and require improvement before implementation. So, if I used their method, and received a cut-score of 40%, that may alert me to the fact that my test is flawed because I am examining a high proportion of unimportant material, and I may wish to alter by test by including more important material (and, if my test matches the learning objectives, then the warning extends to the curriculum). Because it certainly is important for students to be examined on the important material, and setting a test that is both trivial and easy to pass seems to be highly problematic. The authors do address this obliquely with: “Naturally, the more essential content there is in an assessment the higher the cut-score will be. This highlights the need to have the assessment content aligned to the syllabus and aims of the curriculum.” I would, however, like to see an awareness of the dangers of low cut-scores resulting from trivial questions.One smaller issue:The opening paragraph is, well, my goodness ��. What a wall of text. Please don’t do that kind of thing to your reader – I suspect that many readers would simply give up at this stage.:-(.So, an interesting paper, but I would like to see a Version 2 in which the implications of a very low cut-score are discussed. Even if the authors are unmoved on their argument of its importance, they should acknowledge that a too low cut-score may be a warning that something is wrong with the test (and, perhaps, the curriculum/learning objectives). Just what is to be considered a too low cut-score is, however, a difficult point).

Reviewer Expertise:

NA

No decision status is available

# Reviewer report: Reviewer response for version 1

**DOI:** 10.21956/mep.18847.r26863

**Published:** 2021-03-11

**Authors:** Barbara Jennings

**Affiliations:** 1 Norwich Medical School

This review has been migrated. The reviewer awarded 4 stars out of 5 I think this article about standard setting will be of great interest to all assessment leads for undergraduate medical degrees because (as the authors explain) the Angoff approach is the most widely used criterion standard setting (SS) method used in medical schools - and yet I have not found that it is universally loved! The approach is time consuming for the faculty who engage - and it can be hard to get engagement because the underlying philosophy can seem opaque or counter-intuitive to some (in my experience). The authors cite a lot of primary literature in the introduction that may be useful to novice medical educators, and they make a strong argument for the trial that they conducted. The authors propose that Angoff SS can be considered conceptually flawed and describe their rationale for a cross over trial that compares a new SS method designed to emphasise the importance of a performance standard for ALL candidates with the Angoff SS method. The research governance for the study is interesting because all participants in the study, making SS judgements – are also co-authors alongside those responsible for the data analysis. I wonder if the ethics committee chair should have requested a full review & approval to provide better protection and support for researchers and their institutions. The clear rationale for the study and methodology descriptions are definitely strengths e.g. matching of participants in test groups and the well-spaced cross over trial design coupled with a meeting to capture qualitative data that complements the quantitative analyses.One quantitative analysis was for confidence scores about the cut score judgements. The authors’ new alternative method resulted in significantly greater confidence ratings by the participants.Unfortunately, I don’t have the mathematical competence to comment on the validity of the cut-score used in the standard setting methods (perhaps other reviewers will appraise this) but all methods sections are explicitly and clearly described – as are the tabulated results.The authors do not conclude that the new method is statistically better than the Angoff SS, but do show good inter and intra-assessor reliability; they also present findings that suggest it is a philosophically more acceptable and an easier method to apply. The discussion provides a useful account of the strengths and limitations of the methods used and the authors present a helpful cost-benefit summary at the end of the discussion.The authors highlight the importance of clear alignment between curriculum and assessment content (hear hear!) I will definitely be passing a link to the article to our assessment teams and recommend it to others, particularly module leads.

Reviewer Expertise:

NA

No decision status is available
